# Polymer Connectivity Governs Electrophotocatalytic Activity in the Solid State

**DOI:** 10.21203/rs.3.rs-5428587/v1

**Published:** 2024-11-26

**Authors:** Jianheng Ling, Amy L. Vonder Haar, Kiser Z. Colley, Juno Kim, Andrew J. Musser, Phillip J. Milner

**Affiliations:** a Department of Chemistry and Chemical Biology, Cornell University, Ithaca, NY 14853, United States

## Abstract

The reductive functionalization of inert substrates like chloroarenes is a critical yet challenging transformation relevant to both environmental remediation and organic synthesis. Combining electricity and light is an emerging approach to access the deeply reducing potentials required for single electron transfer to chloroarenes, yet this approach is held back by the poor stability and mechanistic ambiguity of current homogeneous systems. Incorporating redox-active moieties into insoluble organic materials represents a promising strategy to unlock new heterogeneous catalytic activity while improving catalyst stability. Herein, we demonstrate the first example of heterogeneous electrophotocatalysis using redox-active rylene diimide polymers for the reduction of chloroarenes. In particular, we find that the electrophotocatalytic activity varies significantly not just as a function of the rylene diimide but also of the redox-inactive polymer backbone. In particular, **PTCDA-en**, a flexible, non-conjugated perylenediimide polymer, outperforms all other tested materials as an electrophotocatalyst. Using transient absorption spectroscopy, we reveal that precomplexation between the closed-shell **PTCDA-en**^**2−**^ and the haloarene substrate is key to productive catalysis. Overall, our work represents the first example of heterogeneous electrophotocatalysis using an insoluble redox-active organic material and provides critical insights into how polymer structure dictates electrophotocatalytic activity in the solid state, guiding the development of next-generation heterogeneous (electro)photocatalysts for sustainable synthesis.

## Introduction

**Figure 1.**
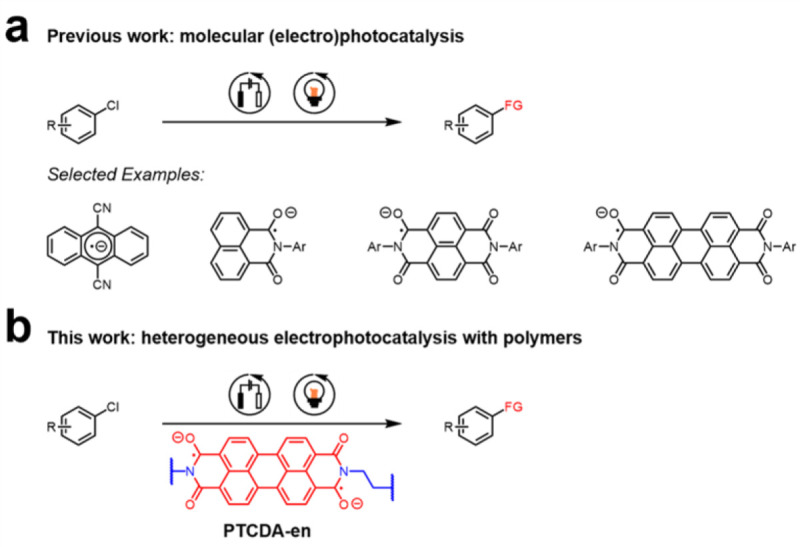
(a) Reductive functionalization of chloroarenes mediated by molecular (electro)photocatalysts. Ar = aryl. (b) Reductive functionalization of chloroarenes mediated by **PTCDA-en**, a perylenediimide (PDI)-based heterogeneous electrophotocatalyst.

Visible light-mediated photoredox catalysis has emerged as a sustainable strategy for constructing carbon–carbon and carbon–heteroatom bonds via single electron transfer (SET).^1–6^ However, many inert yet desirable substrates, such as unactivated chloroarenes, remain difficult to reduce using traditional photoredox catalysts.^7^ In addition, most photocatalytic systems require (super)stoichiometric amounts of terminal reductants to turn over the photocatalyst. The use of electricity in lieu of stoichiometric reagents renders electrochemical reactions more sustainable while providing access to novel reactivity patterns otherwise unattainable using conventional chemistry.^8–13^ Recently, the use of light to excite electrochemically reduced catalysts, termed electron-primed photoredox catalysis or electrophotocatalysis (EPC),^14–21^ has produced deeply reducing photocatalysts that enable the functionalization of inert molecules under milder conditions than direct electrolysis (Figure 1a). However, the active species in many EPC transformations remains ambiguous due to the extremely short photoexcited state lifetimes of the proposed open-shell radical catalytic intermediates.^22^ Additionally, all known EPCs are homogeneous, which compromises their robustness; accordingly, recent work has called the EPC pathway into question due to the facile degradation of proposed intermediates.^23–27^

We propose to overcome these limitations by incorporating EPC-active moieties into insoluble organic materials. Indeed, redox-active materials offer numerous advantages over their homogeneous counterparts, such as robustness and ease of separation from reaction mixtures, while maintaining tunability through structural design.^28–30^ However, redox-active organic materials remain surprisingly underexplored as heterogeneous redox catalysts, likely due to challenges associated with their preparation, characterization, and attachment to electrode surfaces.^30^ Although scattered reports exist regarding catalytic enhancement via the heterogenization of homogeneous catalysts,^31,32^ these approaches suffer from drawbacks such as catalyst instability, limited diffusion in solid supports, and costly immobilization processes.^33^ As such, general strategies to immobilize homogeneous catalysts while preserving or enhancing their catalytic activity remain rare.

Herein, by evaluating a library of redox-active rylene diimide-based polymers, we uncover key structure-property relationships that govern heterogeneous EPC. In particular, we demonstrate that the optimal EPC is a linear polymer in which the redox-active units are electronically isolated by an aliphatic backbone, revealing the unexpected role of polymer connectivity in catalytic activity. Spectroscopic measurements pinpoint that the singlet dianion of the reduced polymer is the catalytically active species, rather than the shorter-lived doublet radical anion. Moreover, we demonstrate that the reaction is driven by the formation of an activated complex between the reduced polymer and the substrate, bypassing the need for long photoexcited state lifetimes for SET to occur.^22^ Taken together, our work not only validates the use of insoluble redox-active organic materials as heterogeneous EPCs but also provides a blueprint for the design of catalysts that can overcome the traditional limitations of photoredox catalysis (Figure 1b).

## Results and discussion

### Materials synthesis and characterization.

Rylene (di)imides were chosen as a model system for achieving EPC in the solid state due to their structural tunability and (electro)chemical stability.^34^ In molecular systems, the photocatalytic activity of reduced rylene (di)imides has been extensively characterized as generally involving a consecutive photoinduced electron transfer (conPET) pathway mediated by stoichiometric reductants.^24,27,35–38^ How these photochemical processes change within a solid is largely unexplored, but previous work with rylene diimide polymers has established the critical influence of the choice of redox-inactive polymer backbone on their redox properties.^39–41^

**Figure 2.**
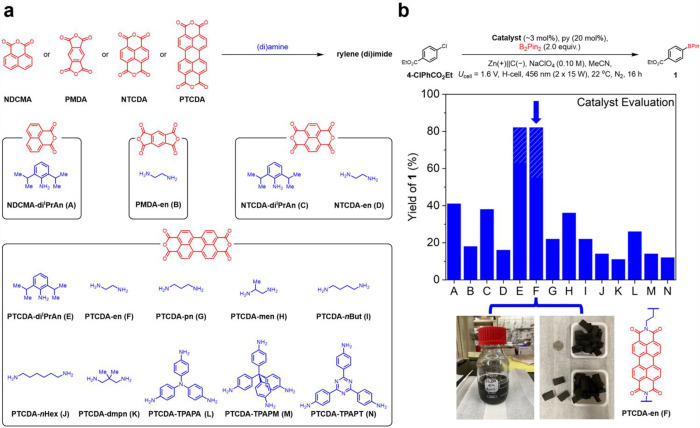
(a) General synthetic scheme of rylene (di)imide-based materials, and structures of (di)anhydrides (red) and amines (blue) used in this work. (b) Model reaction used to evaluate catalyst performance (top), the yield of **1** with different catalysts, showing **PTCDA-en** (**F**) as the best-performing heterogeneous EPC, and bulk production of **PTCDA-en**/PVDF slurry and **PTCDA-en**-functionalized carbon felt electrodes (bottom). The striped regions (**E** & **F**) indicate the yield of **1** obtained from further reaction optimization with the best molecular (**E**) and polymeric (**F**) catalysts.

To probe how structural parameters such as polymer porosity, dimensionality, and flexibility affect EPC activity, we prepared a series of nine perylenediimide (PDI) polymers (Figure 2a). These materials can be readily synthesized from 3,4,9,10-perylene tetracarboxylic dianhydride (**PTCDA**) and amines using molten imidazole as the reaction solvent.^39,40,42^ **PTCDA** was reacted with linear diamines to produce the PDI polymers **PTCDA-en** (**F**), **PTCDA-pn** (**G**), **PTCDA-*n*But** (**I**), and **PTCDA-*n*Hex** (**J**), respectively, with increasing spacer length between PDI units. The polymers **PTCDA-men** (**H**) and **PTCDA-dmpn** (**K**), with increasing steric bulk on the aliphatic backbone, were also synthesized. 2D (**PTCDA-TPAPA** [**L**] and **PTCDA-TPAPT** [**N**]) and 3D (**PTCDA-TPAPM** [**M**]) PDI polymers were also prepared. To test the impact of the rylene diimide subunit on EPC, diimide polymers were also synthesized from 1,4,5,8-naphthalene tetracarboxylic dianhydride (**NTCDA**) or pyromellitic anhydride (**PMDA**) to yield the linear polymers **NTCDA-en** (**D**) and **PMDA-en** (**B**), respectively (Figure 2a). For comparison, we prepared the molecular rylene (di) imides **PTCDA-di**^**i**^**PrAn** (**E**), **NTCDA-di**^**i**^**PrAn** (**C**), and **NDCMA-di**^**i**^**PrAn** (**A**) (Figure 2a, Supporting Information [SI] Section 3). **PTCDA-di**^**i**^**PrAn** exhibits photocatalytic activity via conPET,^38^ whereas **NDCMA-di**^**i**^**PrAn** is described as a potent EPC catalyst under constant current conditions,^17^ although the mechanistic basis for its activity is disputed.^23^

After Soxhlet extraction with organic solvents to remove soluble impurities, the polymers were characterized by attenuated-total-reflectance infrared (ATR-IR) spectroscopy, powder X-ray diffraction (PXRD), diffuse reflectance UV-Vis spectroscopy, thermogravimetric analysis, 77 K N_2_ surface area analysis, cyclic voltammetry (CV), and combustion elemental analysis (SI Section 4). Polyimide formation was established by a consistently observed ~60 cm^−1^ shift in the C=O IR stretching frequency between the dianhydride monomer and the diimide polymer. Combustion elemental analysis and PXRD further support successful polymerization in every case. The Brunauer-Emmett-Teller (BET) surface areas determined from the 77 K N_2_ adsorption data range from nonporous to a high of 396 m^2^/g for **PTCDA-TPAPA**. To assess the electrochemical properties of the polymers, slurries were prepared by mixing 80% active material and 20% polyvinylidene fluoride (PVDF) binder by weight in 1-methyl-2-pyrrolidinone (NMP) followed by deposition onto glassy carbon electrodes. Reversible redox features in the −0.5 to −1 V vs. standard hydrogen electrode (SHE) range were observed for every material in CV measurements, consistent with reversible reduction of the diimides. Together, these characterization data confirm the successful synthesis of a library of redox-active polymers for evaluation as heterogeneous EPCs.

### Reaction development.

The electroreductive radical borylation of ethyl 4-chlorobenzoate (**4-ClPhCO**_**2**_**Et**) to **1** was chosen as a model reaction to benchmark the catalytic performance of the redox-active polymers against their molecular analogs (Figure 2b). This radical coupling provides convenient access to aryl boronates, which are common building blocks in organic synthesis, without the need for precious metal catalysts.^43,44^ Polymer-functionalized cathodes were prepared by vigorously sonicating pre-cut carbon felt electrodes in NMP polymer slurries (80% active material, 20% PVDF) before drying the electrodes under a heat lamp to evaporate residual NMP. Based on the volume of **PTCDA-en** slurry absorbed by the carbon felt, the **PTCDA-en** catalyst loading on the electrode was estimated to be ~3 mol% relative to the chloroarene substrate. The reactions were conducted in H-type divided cells, with bis(pinacolato)diboron (B_2_Pin_2_) as the radical trap, pyridine (py) as an additive to stabilize boryl radical intermediates,^45,46^ sodium perchlorate (NaClO_4_) as the electrolyte, polymer-functionalized carbon felt as the cathode, Zn as the sacrificial anode, and acetonitrile (MeCN) as the solvent (Figure 2b, SI Tables S16–S17). An applied cell potential (*U*_cell_) of 1.6 V was chosen to reduce rylene diimides without deleterious overreduction.^23^ The H-cells were irradiated with blue (456 nm, 2 × 15 W) compact fluorescence lights during electrolysis. Initial evaluation with **PTCDA-di**^**i**^**PrAn** as the catalyst confirmed that both electricity and light are needed to obtain decent yields of **1** (SI Table S18). A comparison of the yields of **1** obtained with various catalysts is included in Figure 2b.

The flexible polymer **PTCDA-en** furnished **1** in the highest yield among the tested polymers, comparable to the molecular analog **PTCDA-di**^**i**^**PrAn** and better than the molecular EPCs **NTCDA-di**^**i**^**PrAn** and **NDCMA-di**^**i**^**PrAn**.^17,19^ Previous work suggests that **PTCDA-en** offers an ideal amount of polymer flexibility to maximize ion diffusion through the polymer matrix while maintaining access to the redox-active PDI sites.^39,40^ Indeed, increasing the steric bulk, flexibility, or rigidity of the polymer backbone all degrade the catalytic performance (Figure 2b). Decreasing the size of the rylene diimide unit while keeping the **en** backbone intact also led to reduced yields of **1**, likely due to the reduced electrochemical stability of these cores compared to PDIs.^39^ Together, these results suggest that *how* the redox-active units are linked is as important as *which* redox-active units are employed to achieve heterogeneous EPC.

Having identified **PTCDA-en** as the optimal polymeric EPC, we further optimized the reaction conditions to improve the yield of the reductive radical borylation to 82% (SI Tables S21–S23; Table 1a, entry 1). After optimization, control experiments revealed that light, **PTCDA-en**, electricity, and py are all necessary to achieve a high yield of **1** (Table 1a, entries 2–5). As a further control, when the reaction was conducted normally for 2 h and then electrolysis was ceased but light irradiation was continued, the yield of **1** did not increase, supporting that continuous electrolysis is necessary to turn over the catalyst (Table 1a, entries 6–7). A much lower yield of **1** was also obtained when **PTCDA-en** was directly added to the reaction mixture in the absence of stirring, rather than grafted onto the carbon felt electrode (Table 1a, entry 8). This finding supports that careful contact between the redox-active polymer and the electrode surface is needed to maximize productive EPC. Notably, the reaction also proceeds smoothly under violet (390 nm) and green (525 nm) light irradiation (Table 1a, entries 9–10). Testing different leaving groups, we found that the corresponding bromoarene, trimethylammonium iodide, and diazonium tetrafluoroborate (Table 1a, entries 11–13) are all competent substrates, albeit less efficient than the corresponding chloroarene.

**PTCDA-en** can be synthesized on > 12 g scale in a single batch, demonstrating its potential scalability (SI Section 4c). The corresponding **PTCDA-en**/PVDF slurry in NMP can also be prepared in bulk, allowing for the fabrication of at least fifty **PTCDA-en**-functionalized carbon felt electrodes in a single batch (Figure 2b). These mass-produced electrodes have similar catalytic activity compared to those produced individually (SI Table S24). Together, these results support that **PTCDA-en** grafted to carbon felt electrodes represents a promising and scalable heterogeneous EPC for the functionalization of diverse arene electrophiles.

### Reaction scope and application.

Evaluating the scope of radical borylation of haloarenes using the optimized heterogeneous conditions (Table 1b), a range of boronate esters (**1a–1d**) were obtained in moderate to good yields. Electron-deficient bromo- and chloroarenes, including those bearing ester (**1–2**), nitrile (**3**), and pyridine (**7**) groups, and the electron-neutral 2-chloronaphthalene (**5**), can be effectively borylated as well. Under more forcing conditions, electron-neutral chloroarenes can be converted into the desired borylated products in moderate to good yields (**4**, **6**).

We hypothesized that leaving out the boryl radical trap under the EPC conditions should allow for the hydrodehalogenation of bromo- and chloroarenes through the formation of aryl anion intermediates (Table 1c). This transformation is useful for the catalytic degradation of halogenated persistent organic pollutants.^47^ Indeed, in the absence of B_2_Pin_2_ and py, electron-deficient and electron-neutral haloarenes can be efficiently hydrodehalogenated (**8–10**) in good yields using **PTCDA-en**. Background reactions conducted in the absence of **PTCDA-en**-functionalized cathodes on selected substrates led to consistently lower yields (SI Table S25), indicating that **PTCDA-en** functions as a heterogeneous EPC in every case.

### Mechanistic investigation.

**Figure 3.**
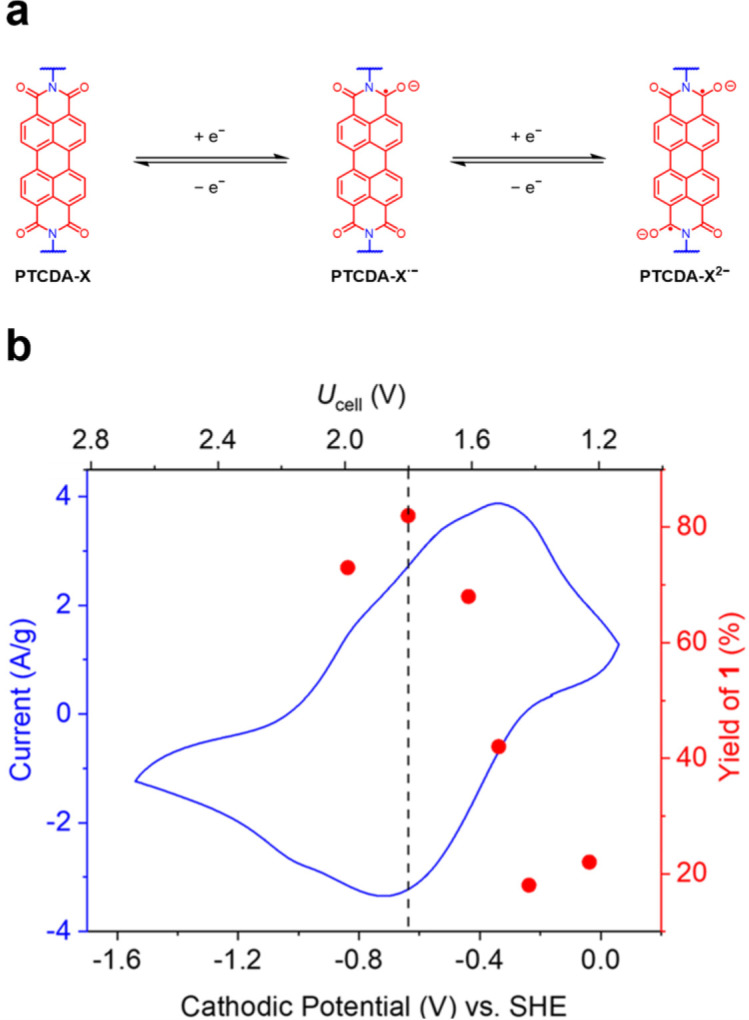
(a) Two one-electron reductions of PDI-based materials. Countercations are omitted for clarity. (b) CV of **PTCDA-en** overlayed with the yield of **1** under various cell potentials (*U*_cell_).

Given the mechanistic ambiguity of previously reported EPC reactions,^17,18,23,24,27^ spectroscopic and electrochemical studies were carried out to probe the catalytically active species and understand why **PTCDA-en** outperforms other polymer catalysts, such as the rigid, conjugated polymer **PTCDA-TPAPA**. Molecular PDIs such as **PTCDA-di**^**i**^**PrAn** are known to undergo reduction to form photochemically active monoanions and dianions (Figure 3a).^35,38,48,49^ Similar to **PTCDA-di**^**i**^**PrAn** (SI Figure S27), **PTCDA-en** can be reduced to **PTCDA-en**^**·−**^ and **PTCDA-en**^**2−**^ (Figure 3b, SI Figure S57).^39^ Using a 3-electrode set-up with a Ag/AgCl reference electrode and a constant Ucell of 1.8 V, the working potential of the **PTCDA-en**-coated cathode was measured to be approximately −0.65 V vs. SHE (SI Figure S166), which is sufficiently negative to reduce **PTCDA-en** to **PTCDA-en**^**2−**^. Using this value as a reference point, the reaction to produce 1 was conducted at a range of Ucell values (1.2–2.0 V); a comparison of the corresponding reaction yields indicates that the yield maximizes at sufficiently negative cathodic potentials to produce **PTCDA-en**^**2−**^ (−0.5 to −0.8 V, Figure 3b).

**Figure 4.**
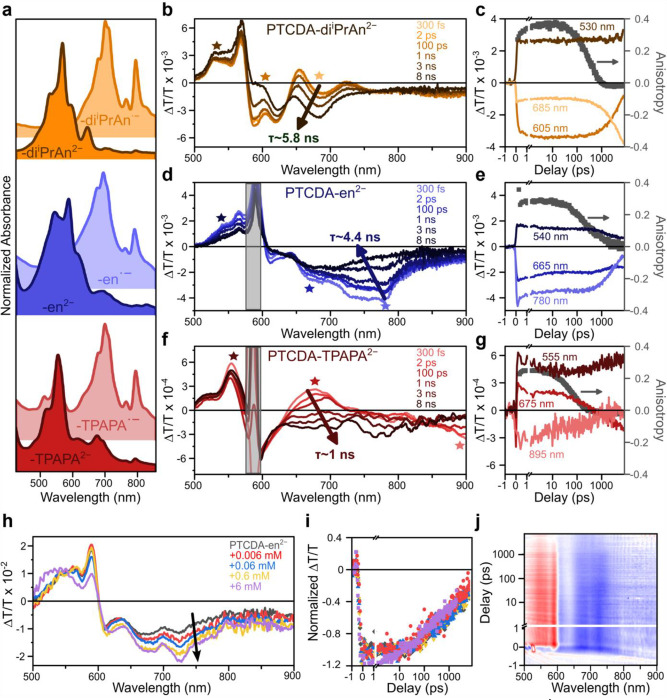
(a) Normalized steady-state absorption spectra of chemically reduced **PTCDA-di**^**i**^**PrAn, PTCDA-en**, and **PTCDA-TPAPA** in DMF. TA spectra of (b) **PTCDA-di**^**i**^**PrAn**^**2−**^, (d) **PTCDA-en**^**2−**^, and (f) **PTCDA-TPAPA**^**2−**^ at select time delays following excitation at 585 nm. Arrows highlight the primary conversion kinetics into a long-lived species beyond the temporal range of the instrument, and strong pump scatter due to limited solubility is covered by grey boxes. (c) (e) (g) Decay kinetics for the probe wavelengths indicated by stars in in the corresponding TA spectra, with anisotropy decay (grey squares). All anisotropy data was acquired following excitation at 456 nm. (h) TA spectra extracted 2 ps after photoexcitation for a neat solution of photochemically doped **PTCDA-en**^**2−**^ in DMF (grey) and equivalent solutions mixed with increasing concentrations of **4-ClPhCO**_**2**_**Et**. Spectra are normalized by the area of ground-state bleaching (ΔT/T > 0) to standardize the effective photoexcited population. Arrow is a guide to the eye. All samples were excited at 456 nm. (i) Integrated normalized TA kinetics of the photoinduced absorption across the quenching series. (j) Full TA dataset for 0.06 mM **4-ClPhCO**_**2**_**Et**. The photoproduct signal (blue features > 600 nm) is present from the instrument response of ~200 fs.

Because the CV features corresponding to the **PTCDA-en**/**PTCDA-en**^**·−**^ and **PTCDA-en**^**·−**^/**PTCDA-en**^**2−**^ redox events are not cleanly resolved (Figure 3b), further spectroscopic studies were undertaken (Figure 4). Although the reduced polymers are insoluble under the reaction conditions, UV-Vis spectroscopy confirms that PTCDA-di^i^PrAn can be cleanly reduced to **PTCDA-di^i^PrAn^·−^** and **PTCDA-di^i^PrAn^2−^** in *N*,*N*-dimethylformamide (DMF) using tetrakis(dimethylamino)ethylene (TDAE),^50^ a potent organic reductant. After confirming that this process yields the same species with the same photophysical behavior as electrolysis (SI Figure S181) or chemical reduction (SI Figure S195) in MeCN, TDAE was employed to reduce the polymer EPCs **PTCDA-en** and **PTCDA-TPAPA**. Both materials readily dissolve in DMF upon reduction, affording spectroscopic access to **PTCDA-en**^**·−**^ (SI Figure S197), **PTCDA-en**^**2−**^ (SI Figure S199), **PTCDA-TPAPA**^**·−**^ (SI Figure S205), and **PTCDA-TPAPA**^**2−**^ (SI Figure S207). By UV-Vis spectroscopy, the monoanions of reduced **PTCDA-di**^**i**^**PrAn**, **PTCDA-en**, and **PTCDA-TPAPA** dissolved in DMF exhibit remarkably similar features, as do the corresponding dianions (Figure 4a). We observe the same characteristic series of electronic and vibronic bands,^51–53^ with **PTCDA-en** red-shifted and **PTCDA-TPAPA** blue-shifted relative to **PTCDA-di**^**i**^**PrAn**. This suggests that the reduced PDI units are relatively isolated in both the non-conjugated (**PTCDA-en**) and conjugated (**PTCDA-TPAPA**) materials. As such, changes in the ground-state electronic structures are insufficient to explain the marked difference in reactivity (Figure 2b, F vs. L).

Transient absorption (TA) spectroscopy was used to gain deeper insight into the photophysics of **PTCDA-di**^**i**^**PrAn**^**2−**^, **PTCDA-en**^**2−**^, and **PTCDA-TPAPA**^**2−**^ (Figure 4b–g) and the corresponding monoanions (SI Figures S211–S212 & S214). In these pump-probe measurements, signals with ΔT/T > 0 represent bleaching of the ground-state absorption of the excited molecules or stimulated emission from bright excited states, whereas signals with ΔT/T < 0 correspond to photoinduced absorption and reveal the fingerprints of distinct electronic states. In **PTCDA-di**^**i**^**PrAn**^**2−**^ (Figure 4b–c), a conversion from the initial singlet (orange) to a metastable species (brown) with a lifetime of ≪ 8 ns can be resolved. The latter spectrum has previously been attributed to a triplet state.^53^ It also bears resemblance to **PTCDA-di**^**i**^**PrAn**^**·−**^ (namely, the strong absorption ~700 nm), potentially suggestive of photoinduced **PTCDA-di**^**i**^**PrAn**^**2−**^ → **PTCDA-di**^**i**^**PrAn**^**·−**^ conversion. A timescale of 5.8 ns was recovered for this conversion, consistent with previous studies and significantly longer than the 160 ps lifetime of photoexcited **PTCDA-di**^**i**^**PrAn**^**·−**^ (SI Figure S211).^48,51–53^ Together, these findings point to the greater photochemical accessibility of **PTCDA-di**^**i**^**PrAn**^**2−**^ compared to **PTCDA-di**^**i**^**PrAn**^**·−**^.^49^

Turning to the reduced polymers, **PTCDA-en**^**2−**^ exhibits similar spectral features to **PTCDA-di**^**i**^**PrAn**^**2−**^ (Figure 4d–e). The principal difference in excited-state spectra is the stronger photoinduced absorption in **PTCDA-di**^**i**^**PrAn**^**2−**^, which can be attributed to a red-shift that reduces overlap between this negative feature and the positive ground-state bleach. A relatively slow transition from the singlet (blue) into a long-lived metastable species (black) can again be resolved, with a final absorption fingerprint that compellingly points to **PTCDA-en**^**2−**^ → **PTCDA-en**^**·−**^ conversion. The timescale of this process (~4.4 ns) agrees well with that of **PTCDA-di**^**i**^**PrAn**^**2−**^, suggesting that polymerization has not strongly perturbed the excited-state electronic structure in **PTCDA-en**. In contrast to the short-lived excitation of **PTCDA-en**^**·−**^ (~190 ps, SI Figure S212), **PTCDA-en**^**2−**^ is more photochemically viable, though it is unclear which of the two species in Figure 4e engages in SET to the chloroarene substrate.

The TA spectra of **PTCDA-TPAPA**^**2−**^ provide the first insights into the worse catalytic performance of this material (Figure 4f–g). While a two-state progression from a recognizable singlet (red) to a long-lived (black) state is again observed, the final species bears no resemblance to that observed in **PTCDA-di**^**i**^**PrAn**^**2−**^ or **PTCDA-en**^**2−**^, nor does it carry any characteristic features of **PTCDA-TPAPA**^**·−**^. These observations point to the availability of new decay pathways due to the conjugated backbone linking the PDI units in **PTCDA-TPAPA**, hence the faster timescale of conversion (~1 ns) and reduced magnitude of long-lived signal in this material. These findings suggest the key to efficient heterogeneous EPC is to isolate the catalytically active PDI^2−^ states from one another to minimize unproductive decay pathways, as in **PTCDA-en**.

Crucial insight into the differences between the polymer catalysts can be gleaned through transient anisotropy measurements (grey squares in Figure 4c, e, g). The anisotropy decay of **PTCDA-di**^**i**^**PrAn**^**2−**^ was found to be ~250 ps, which is distinctly faster than the electronic kinetics, allowing assignment to physical rotation of the molecule (Figure 4c). Given that polymeric **PTCDA-en**^**2−**^ and **PTCDA-TPAPA**^**2−**^ are more massive, 250 ps should be a lower limit for any rotational effects. The corresponding anisotropy decays in both reduced polymers are multiexponential, reflecting the larger disorder in these systems, with amplitude-weighted average lifetimes of 544 ps for **PTCDA-en**^**2−**^ (Figure 4e) and 133 ps for **PTCDA-TPAPA**^**2−**^ (Figure 4g). The excitation in **PTCDA-en**^**2−**^ appears to remain on the same site out to the nanosecond timescale, pointing to minimal coupling between neighboring sites. We thus conclude that the flexible, non-conjugated backbone in **PTCDA-en**^**2−**^ largely preserves the favorable electronic structure of **PTCDA-di**^**i**^**PrAn**^**2−**^. In contrast, the anisotropy decay in rigid, conjugated **PTCDA-TPAPA**^**2−**^ reveals a relatively fast site-to-site hopping timescale, a consequence of much stronger interactions between nearby PDI^2−^ sites. These interactions introduce new decay pathways and alter the nature of the long-lived electronic state, compromising photocatalytic activity.

Although TA measurements provide rich insight into relative catalytic performance, they do not provide a definitive answer regarding which electronic state is involved in EPC. Early reports of photocatalytic conversion involving excited radical anions have met with controversy due to the short excited-state lifetimes (typically ≪ 1 ns) of these open-shell species.^18,22–25,27,38,48^ Alternative mechanisms involve photo- or electrochemical side products that are closed-shell and thus exhibit much longer (> 1 ns) lifetimes,^23,27^ like the dianions studied here. However, at sufficiently high substrate concentrations, dynamic quenching has been observed even for the short-lived excited **PTCDA-di**^**i**^**PrAn**^**·−**^,^48^ meaning either **PTCDA-en**^**·−**^ or **PTCDA-en**^**2−**^, or either excited state of **PTCDA-en**^**2−**^, may engage in catalysis.

**Figure 5.**
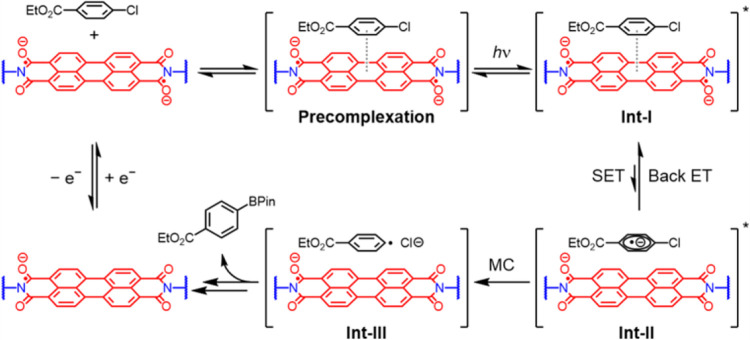
Proposed mechanism of photoinduced SET from **PTCDA-en**^**2−**^ to **4-ClPhCO**_**2**_**Et**. MC denotes mesolytic cleavage. Countercations and B_2_Pin_2_ are omitted for clarity.

To distinguish among these possibilities, we performed quenching assays with solutions of **PTCDA-en**^**2−**^ dissolved in DMF, varying the concentration of **4-ClPhCO**_**2**_**Et** from 0.006 mM to 6 mM. The same basic TA spectral features are observed in the presence and absence of **4-ClPhCO**_**2**_**Et** (Figure 4h). Furthermore, the TA kinetics reveal that the dynamics are entirely independent of **4-ClPhCO**_**2**_**Et** concentration over 3 orders of magnitude (Figure 4i). Increasing **4-ClPhCO**_**2**_**Et** concentration only increases the relative weight of the photoinduced absorption centered around 700 nm (arrow in Figure 4h). This quencher-related band closely matches the ground-state absorption of **PTCDA-en**^**·−**^; that is, the formation of **PTCDA-en**^**·−**^ directly correlates with increased **4-ClPhCO**_**2**_**Et** concentration. This spectral signature is direct evidence that **4-ClPhCO**_**2**_**Et** is reduced by photoexcited **PTCDA-en**^**2−**^. However, the lack of dynamic quenching reveals that neither excited state reported in Figure 4d is directly involved in the reaction. Indeed, this photoproduct is detected and achieves its maximum amplitude already within the 200-fs resolution of the experiment (Figure 4j). Instead of a dynamic mechanism, photoinduced SET must occur within an activated complex between **PTCDA-en**^**2−**^ and **4-ClPhCO**_**2**_**Et** (**Int-I**, Figure 5), essentially immediately upon photoexcitation. The role of such precomplexation has been suggested to explain the anomalous apparent potency of open-shell molecular (electro)photocatalysts,^22,54^ yet, to our knowledge, this is the first direct evidence for precomplexation in a closed-shell EPC.

The importance of precomplexation becomes especially clear when considering the reaction behavior inferred from Figure 4i. Even at high **4-ClPhCO**_**2**_**Et** concentrations, the fingerprint of photoproduct **PTCDA-en**^**·−**^ decays with a lifetime effectively indistinguishable from the **4-ClPhCO**_**2**_**Et**-free experiment, nor is significant build-up of permanent photoproducts observed. These observations point to a low yield of reaction per photon absorbed, despite the facile initial SET from **Int-I** to **Int-II** (Figure 5). There is a significant driving force for back ET to regenerate **PTCDA-en**^**2−**^ (**Int-I**), which can only be prevented by mesolytic cleavage (MC) of the C–Cl bond, leading to the formation of aryl radical (**Int-III**) and ultimately the desired borylated product (Figure 5). On the surface, this is an unfavorable setup for productive photochemistry and one that should be common to any such highly energetic photocatalytic conversions. However, precomplexation between **4-ClPhCO**_**2**_**Et** and **PTCDA-en**^**2−**^ can keep the reactive pair together for multiple cycles of photoexcitation, with the quasiequilibrium established by continuous irradiation only broken by MC.

Overall, these spectroscopic studies highlight that: 1) the closed-shell PDI^2−^ is more photochemically viable compared to the open-shell PDI^·−^, 2) the flexible, non-conjugated **PTCDA-en**^**2−**^ contains electronically isolated PDI^2−^ sites that resemble those of **PTCDA-di**^**i**^**PrAn**^**2−**^, which accounts for its catalytic competence (Figure 2b), and 3) that an activated complex between **PTCDA-en**^**2−**^ and the chloroarene substrate (**Int-I**, Figure 5) is likely key to effective heterogeneous EPC.

## Conclusion

Employing a user-friendly strategy to graft redox-active polymers onto carbon felt electrodes, we evaluate a library of rylene diimide polymers for their efficacy in heterogeneous EPC. The best-performing polymer catalyst, **PTCDA-en**, possesses redox-active PDI units linked by flexible, non-conjugated aliphatic linkages, mimicking the behavior of the molecular catalyst in an extended material. Through in-depth electrochemical and spectroscopic studies, we identify the closed-shell dianion **PTCDA-en**^**2−**^ as the catalytically active species that undergoes photoinduced SET to haloarene substrates via precomplexation (**Int-I**, Figure 5).

Critically, our findings reveal a key structural design principle underpinning the construction of heterogeneous EPCs: material connectivity matters. It is not enough to simply incorporate redox-active moieties into any polymeric material, as the redox-inactive polymer backbone plays a significant role in EPC activity. An appropriate amount of polymer flexibility maximizes ion diffusion, ensures access to redox-active sites within the polymer, and isolates the redox-active sites to maximize their catalytic activity. Moreover, our results highlight a straightforward approach to harness the reactivity of short-lived excited states through activated complex formation. Such complexes circumvent the low per-photon efficiency intrinsic to these energetically challenging reactions by maintaining the substrate-catalyst pair for multiple excitation cycles. This behavior has the potential to be readily programmed into modular materials, providing a design principle for the use of extremely short-lived reactive species for catalysis.

## Figures and Tables

**Table 1.** Reaction development.

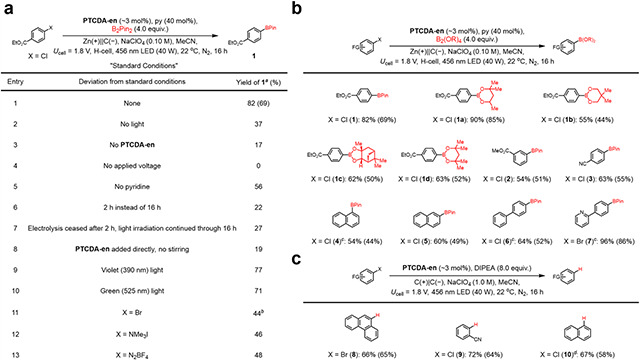

(a) Optimization of model reaction.

a Yields determined by ^1^H NMR with CH_2_Br_2_ as the internal standard. Isolated yields in parentheses.

b 8 equiv. B_2_Pin_2_, 80 mol% py.

(b) Scope of **PTCDA-en**-catalyzed reductive borylation.

c 2 equiv. py, NaClO_4_ (1.0 M).

(c) Scope of **PTCDA-en**-catalyzed reductive dehalogenation.

d No DIPEA, H-cell was used, Zn(+)||C(−).
